# Development and validation of an app-based mindfulness program for older adults: A randomized controlled trial

**DOI:** 10.1093/geroni/igaf122.4303

**Published:** 2025-12-31

**Authors:** Shuting Dai, Yahui Wang, Dannii Yeung

**Affiliations:** City University of Hong Kong, Hong Kong, China (People’s Republic); City University of Hong Kong, Hong Kong, China (People’s Republic); City University of Hong Kong, Hong Kong, China (People’s Republic)

## Abstract

Objectives Despite the growing popularity of digital mindfulness-based interventions (MBIs), existing programs often vary in duration and format, and few are tailored for older adults. This study aimed to develop and validate a 4-week, app-based mindfulness program tailored for older adults. Methods Using a randomized controlled trial design, a total of 240 older Chinese adults were recruited and randomized into either a 4-week app-based mindfulness program (MF; n = 120) or a digital health education program (HE; n = 120). Outcome variables include depressive symptoms and state mindfulness level. Results Preliminary analyses included participants who completed baseline (T0) and post-intervention (T1) assessments (MF: n = 46; HE: n = 54), with follow-up (T2) data available for a smaller subset (MF: n = 11; HE: n = 13). At T0, the two groups did not differ significantly on depressive symptoms (MF: M = 7.07, SD = 1.97; HE: M = 6.78, SD = 1.74) and state mindfulness (MF: M = 59.35, SD = 5.13; HE: M = 60.31, SD = 5.64). Mixed-effects models from T0 to T2 showed significant group (2) × time (3) interactions for depressive symptoms (F (2, 134.52) =3.95, p=.019, ηp²=.055) and state mindfulness (F (2, 128.69) =3.42, p=.032, ηp²=.050). Conclusions Preliminary findings indicate that the 4-week, app-based mindfulness program is feasible and can reduce depressive symptoms and improve state mindfulness level in older adults. These results suggest that digital mindfulness intervention is an accessible approach to support mental health in late adulthood.

